# Clinical Lectures on Neurasthenia

**Published:** 1900-04

**Authors:** 


### Book Reviews.

Clinical Lectures on Neurasthenia. By Thomas D. Savill, M.D., Physician to The West-End Hospital for Diseases of the Nervous System. Pp. 144. London, etc. New York : William Wood & Co. 1899.

The lectures, five in number, were collected and published separately at the solicitation of several of the author's friends, who found in them valuable ideas and experience worthy of a better fate. The first of these lectures was delivered as part of the post-graduate course, which was organised at the Paddington Infirmary in the year 1891. All the other lectures were delivered at the Willbeck Street Hospital for diseases of the nervous system during the years 1897-8, and formed part of the post-graduate course of lectures, delivered by different members of the staff. Among the author's reasons for acceding to the request were the wealth of material to chose from the unjustifiably hopeless view taken by the profession concerning the curability of diseases of the nervous system in general and of the so-called functional disorders in particular and the scarcity of knowledge in the current text-books in regard to the treatment with the various trivial symptoms presented by these patients. As a result of this, not only do many curable patients go unrelieved, but a good number have recourse to charlatans.

In chapter I. the author describes the infirmary and depicts its advantages to be gained by the clinical observer in such a place. He then describes the five methods of research: (1) embryological, (2) secondary degeneration, (3) animal experimentation, (4) the anatomo-clinical method, and (5) the method of analogy; this last method being especially applicable to diseases of a functional nature. Then follows a carefully prepared scheme for making the examination of the patient and a very able outline of the pathology of functional disorders of the nervous system. The author believes that these disorders are due in one of six different ways: (1) by the agencies which have a pathological foundation, but which as yet are undiscovered. Included in this group are placed paralysis, agitans, senile tremor and the various spasms; (2) those profound alterations of function, due to toxic substances introduced into the body from without; (3) alteration of function may take place by toxic substances manufactured within the body; 4 alteration of function where the nutrition of the nerve tissues is at fault; (5) those disturbances due to either a deficiency or excess in the quantity of blood flowing to a part without necessarily any attention in its quality, and lastly there is another group of functional disorders of the nervous system due to an increased reflex irritability, or an increased response to impressions from without. The author very wisely impresses upon his hearers that the time has gone by when everything functional, everything non-structural can be called by the name hysteria.

Lecture II. is devoted to the study proper of nervousness or neurasthenia. It is but a short time ago that the idea of neurasthenia being a clinical entity was scouted by English writers, and scarcely referred to in the leading text-books. Dr. Savill in this chapter proves himself to be a firm believer in the doctrine that neurasthenia is not hysteria on the one hand nor hypochondria on the other, but "an irritable weakness of the entire nervous system, characterised by hypersensitiveness of the tactile sensibility and special senses; by headache, inaptitude for mental work, disturbed sleep and irritability of temper (when the brain is chiefly affected); by general weakness, restlessness, nervousness, and vague pains (when the spinal cord is chiefly affected and usually accompanied (in both forms) by various phenomena referable to the vaso-motor and sympathetic systems." The author then gives examples of cases of this affection, each case selected to illustrate some point in diagnosis, or in symptomatology. The author makes the statement that 90 per cent. of all cases coming to him for mere nervousness are suffering with neurasthenia.

Regarding the symptoms the author states that nearly all the symptoms are subjective, and that the only objective ones are the exaggerated knee jerks and ocular symptoms. The whole general bearing of the case, the tremor, the cornea, the mouth, are all objective symptoms and are perhaps better set forth in our American than in the English cases.

In Lecture III. the author takes up very carefully the question of diagnosis of neurasthenia and the relation between neurasthenia and gastric disorders. These cases, the author believes are due to toxic blood conditions. Out of 157 private cases 74 revealed definite evidences of some form of dyspepsia, and the writer asserts with much reason that gastric disorders result in a defective elaboration of the products of digestion, and the pouring into the blood of a large quantity of imperfectly elaborated and toxic products. Constipation is capable of acting detrimentally in the same way owing to the reabsorption of many materials which are intended for excretion. The author states most positively that neurasthenia is consequent to gastric and intestinal disorders and goes to considerable pains in elaborating upon the different views held by various authors on this much mooted question. Cases are also cited to sustain the author's opinion and his arguments are convincing.

In lecture IV. the author takes up the various forms of neurasthenia; its rapid increase and reasons; the treatment indicated in the several groups and the importance of detail in the care of these cases.

In lecture V. are considered the mental symptoms of neurasthenia and neurasthenic insanity. Of the latter the author states there are three possible events, which in order of frequency are as follows: (1) Recovery—about nine-tenths of the author's cases have terminated this way after an illness of months; (2) In a few cases, all of which have been advanced in years, the mind fails to recover; all the faculties slowly deteriorate, and a condition of dementia gradually supervenes; (3) death may occasionally ensue, either from progressive asthma, or, as is most common, from a low form of pneumonia.

In the addendum, prepared by Agnes Blackadder, the author gives the various names which have been applied by various authors to conditions resembling neurasthenia, and the opinion of various authors in regard to the nature and origin of traumatic neurasthenia, together with a very full bibliography on the subject, in which full credit is given to Beard for his early and exhaustive writings. The book is well printed, on excellent paper and substantially bound.

